# Protocol for comparing two training approaches for primary care professionals implementing the Safe Environment for Every Kid (SEEK) model

**DOI:** 10.1186/s43058-020-00059-9

**Published:** 2020-09-22

**Authors:** Howard Dubowitz, Lisa Saldana, Laurence A. Magder, Lawrence A. Palinkas, John A. Landsverk, Rose L. Belanger, Ugonna S. Nwosu

**Affiliations:** 1 Division of Child Protection, Department of Pediatrics, University of Maryland School of Medicine, 520 W. Lombard St, Baltimore, MD 21201 USA; 2 Oregon Social Learning Center, 10 Shelton McMurphey Blvd., Eugene, OR 97401 USA; 3 Department of Epidemiology and Public Health, University of Maryland School of Medicine, 655 W. Baltimore St., Baltimore, MD 21201 USA; 4 Suzanne Dworak-Peck School of Social Work, University of Southern California, 669 W. 34th Street, MC0411, Los Angeles, CA 90089-0411 USA

**Keywords:** Pediatrics, Family medicine, Primary care, Prevention, Child maltreatment, Social determinants of health, SEEK

## Abstract

**Background:**

Child maltreatment (CM) is a major public health problem, affecting many lives, in the short and long term, and costing individuals, families, and society dearly. There is a need for broad implementation of evidence-based preventive interventions, such as the Safe Environment for Every Kid (SEEK) model, developed for pediatric primary care. Primary care offers an excellent opportunity to help address prevalent psychosocial problems (e.g., parental depression) that are risk factors for CM. By addressing such problems, SEEK can strengthen families and support parents; promote children’s health, development, and safety; help prevent CM; and benefit the health of the US population. This study will examine intervention strategies for optimizing SEEK’s adoption, implementation, and sustainment, and its effectiveness in preventing CM.

Despite strong evidence from two federally funded randomized controlled trials, SEEK has not been widely adopted. The goal of this study is to examine technology-driven implementation strategies to scale-up SEEK—in pediatric and family medicine primary care settings. The aims are to (1) evaluate the effectiveness of training strategies on SEEK’s implementation in primary care practices, (2) evaluate barriers and facilitators to successful implementation and sustainment of SEEK, and (3) examine the model’s effectiveness in preventing CM and the economic costs of implementing SEEK.

**Methods:**

This randomized type III hybrid mixed methods design will examine how advances in medical training can bolster SEEK’s adoption and implementation in pediatric and family medicine practices in different regions of the USA. These are independent online training and in-depth structured training via a quality improvement project, approved by the American Boards of Pediatrics and of Family Medicine. We will also evaluate SEEK*online*, software that assists primary care practitioners implement the model, and a “Traditional” paper and pencil strategy for their impact on implementation. The study uses the EPIS framework and the Universal Stages of Implementation Completion, quantitative measures, qualitative interviews, and data abstracted from electronic health records.

**Discussion:**

The knowledge gained should improve pediatric primary care to better address prevalent social determinants of health, benefiting many children and families. The outcomes should enhance the field of implementation science and guide future interventions in primary care.

**Trial registration:**

NCT03642327, Clinical Trials, registered August 21, 2018.

Contributions to the literature

- The National SEEK Study will guide the adoption, implementation, and sustainment of this evidence-based model to prevent child maltreatment, in pediatric and family medicine primary care practices.
- This hybrid III study will examine the effectiveness of the SEEK model in preventing child maltreatment, and its associated economic costs.
- This study will provide useful information on technology-driven strategies for training medical professionals and for introducing innovations into healthcare delivery.

## Background

Child maltreatment (CM) is a major public health problem in the USA, affecting many lives, in the short and long term, and costing individuals, families, and our society dearly. In 2017, 7.5 million children were reported to Child Protective Services (CPS) [1]. Of these, 674,000 children (i.e., 9.2 per 1000) were “substantiated” victims of CM. Yet, reported cases capture only the tip of the iceberg. The National Incidence Study-4, using observations by community professionals, estimated that 1.26 million children (i.e., 17.1 per 1,000) were maltreated in 2005–2006; the more inclusive “Endangerment Standard” estimated nearly 3 million victims (i.e., 40 per 1,000) [2].

The consequences of CM can be devastating. In addition to injuries and physical health problems, child and adolescent sequelae include many psychological and behavioral problems [3–7]. CM has also been linked to an array of adult outcomes such as substance use disorders, HIV/AIDS-related sexual risk behaviors [8–14], and intimate partner violence (IPV) [15–20], as well as depression, suicide, criminal behavior, interpersonal problems, academic and vocational difficulties [21–30], and multiple physical health problems [31–34]. The financial costs of CM are immense. Two thirds of the medical costs of CM are paid through Medicaid [35]. Additional costs are incurred by the child welfare, educational, mental health, and judicial systems, with estimated US costs of $103.8 billion per year [36]. The human and economic costs of CM point to the need for effective preventive strategies and scaling up evidence-based practices (EBPs).

**Fig. 1:**
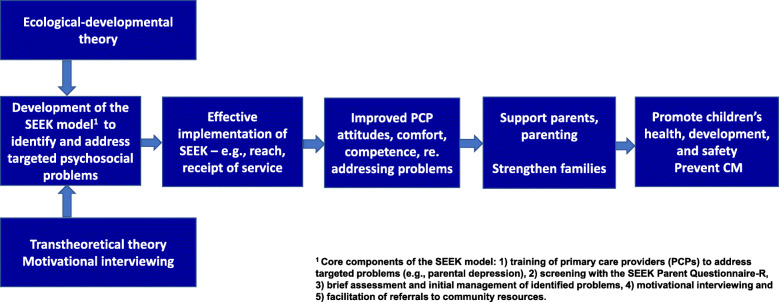
Conceptual model for SEEK

Despite the compelling need to prevent CM, few interventions other than home visiting programs have been rigorously evaluated and found to be effective. Further, programs have not been developed for the healthcare system with the exception of preventing abusive head trauma, an important but small component of CM. Another is the Safe Environment for Every Kid (SEEK) model, developed for pediatric primary care, and found to prevent CM in two large federally funded, randomized controlled trials (RCTs) [37–40]. The conceptual basis underpinning the model is shown in Fig. 1. Several theories guided SEEK’s development. Ecological-developmental theory recognizes the multiple and interacting systems surrounding a child [41, 42]. Pediatric care has mostly focused narrowly on the child; SEEK was based on understanding the influence of family and parental functioning on children’s health, development, and safety and on CM. SEEK also was guided by the transtheoretical model, linking an understanding of a person’s stage of change (e.g., pre-contemplative) with interventions tailored to the individual [43–45]. Principles of motivational interviewing (MI) have been incorporated [46, 47]. Prevention science, integrating multiple disciplines, also guided SEEK’s development [48]. CM, with its multifactorial etiology, demands collaboration among disciplines. SEEK aims to enhance primary care professionals’ (PCPs’) abilities to address targeted social determinants of health (SDH), working with professionals in other disciplines and agencies. The SEEK model was also guided by social cognitive theory [49]. For example, role plays demonstrate how PCPs can help address problems. In addition, the US Preventive Services Task Force recommends screening for intimate partner violence (IPV), depression, and alcohol misuse [50]. SEEK provides a structured approach to follow these recommendations.

Core components of the SEEK model include the following: (1) *training PCPs* to identify and help address targeted SDH that are also risk factors for CM—parental depression and major stress, substance abuse, IPV, harsh discipline, and food insecurity; (2) the evidence-based *SEEK Parent Questionnaire-R* (PQ-R) to screen for the problems at well child visits [51–56]; (3) the Reflect–Empathize–Assess–Plan (REAP) approach to help PCPs assess and address problems; (4) principles of MI; (5) *SEEK Parent Handouts* for targeted problems, customized with local resources; and (6) referrals to community resources.

## Preliminary effectiveness of SEEK

There is good evidence supporting SEEK based on two RCTs (SEEK I and II) [37–40]. The SEEK I sample of 558 families was high risk, very low income, urban, mostly African American and served by pediatric resident clinics in Baltimore; it involved 95 physicians [37–40]. SEEK II involved 105 pediatricians and nurse practitioners and 1119 relatively low risk, mostly white, middle-income families recruited from 18 suburban private pediatric practices in central Maryland [38, 39].

Practices in both RCTs were randomized to either SEEK or standard care. Following baseline evaluations, PCPs randomized to SEEK received in-person training on addressing the targeted SDH; they were evaluated at 18–36 months after the initial training. Parents were recruited from all practices with initial and follow-up assessments at 6 and/or 12 months. Toward the end of the studies, after 30–43 months (SEEK I and II), the children’s medical records were reviewed for CM-related diagnoses, before and after implementing SEEK. Data were gathered from the state agency on possible CPS involvement. We assessed parents’ satisfaction with the child’s PCP. In SEEK II, medical students observed PCPs during 3 checkups, at baseline and at study end, to observe their approach to and time spent on the targeted problems.

### Impact on PCPs

In both studies, PCPs in SEEK practices reported significantly greater comfort and perceived competence in addressing the targeted problems, compared to controls [37–40]. Improvements were sustained for 18–36 months. Review of medical records revealed that SEEK PCPs were more likely than controls to screen for the targeted risk factors for CM. In SEEK II, this was confirmed by direct observation; screening increased on average across practices from less than 5 to 62% of visits. In SEEK I, parents in SEEK clinics reported more favorable views of their child’s PCP [40]. Importantly, busy PCPs demonstrated they could effectively implement SEEK.

### Impact on CM

Three measures from three sources assessed CM:

*Parent self-report.* SEEK I parents reported fewer “severe physical assaults” than controls (0.11 vs. 0.33, *p* = .04) [37]. SEEK II parents reported fewer instances of psychological aggression (*p* = .02) and minor physical assaults (*p* < .05) than did controls [38].

*Medical records.* Children in SEEK I practices had less medical neglect than did controls [9]. There was less “non-compliance” with medical care (4.6% vs. 8.4%, *p* = 0.05) and fewer delayed immunizations (3.3% vs. 9.6%, *p* = 0.002).

*CPS reports.* In SEEK I, fewer families were reported to CPS (12% vs. 19.7%, *p* = .04) [9]. A report was prevented in one of every 13 such families exposed to SEEK.

### Time required

SEEK did not require significantly more time, on average, for PCPs to address psychosocial problems [39]. Parents completing the PQ-R before visits saved time; this was offset when addressing problems.

### Cost

SEEK II cost $3.38 per child per year and $306 per CM experience prevented. Using a conservative estimate of the healthcare cost per case of CM at $2779, providing SEEK in all practices would have saved society $2,151,878 for 29,610 children [57]. Thus, SEEK has a positive cost benefit. However, it does not necessarily follow that practices can afford to implement SEEK. The current study focuses on examining the costs of implementing the model.

The strong evidence for SEEK’s effectiveness has been underscored by its listing on the websites of the US Centers for Disease Control and Prevention, the Agency for Healthcare Research and Quality, the American Academy of Pediatrics, and by the California Clearinghouse for Evidence-Based Interventions in Child Welfare. Early adopters are increasingly implementing SEEK in primary care settings, including in Sweden. SEEK’s adoption by primary care clinics has, however, been limited, and questions remain as to how best implement this model. Given that early adopters are estimated to be 10% of those eligible and that medical innovations can take 17 years to be adopted, it is unlikely that SEEK’s potential public health benefit will be realized without better methods for scaling up [58]. To help facilitate its implementation, two pragmatic technology-driven platforms have been developed to increase the convenience and accessibility of the model to busy PCPs: (1) an interactive web-based training that utilizes webinars and individualized guidance during start-up and (2) SEEK*online* software to facilitate delivery of SEEK within regular checkup visits. This study will examine the effectiveness of these implementation strategies.

### Training strategies

Two training strategies will be tested (see Table 1), both increasingly used with PCPs, and developed in accordance with principles of adult leaning [59–64]. First, SEEK Maintenance of Certification (MOC-4**)** is an example of a structured quality improvement (QI) project approved by the American Boards of Pediatrics and of Family Medicine, required of physicians to maintain Board certification. It includes viewing the training videos and implementing SEEK in one’s practice and conducting the QI Plan-Do-Study-Act (PDSA) cycle. This PDSA cycle involves learning from data collected before and during SEEK’s implementation to assess and improve the process [65]. Four 1-hr webinars over 4 months enable collaborative learning and mentoring. Second, independent online training (IND) is a student-centered approach; it involves viewing the training videos over 2–3 hrs and passing the post-test. Both approaches include three 1-hr consultations in the ensuing year and offer CME credits toward state licensure. We hypothesize that the interactive MOC-4 training will lead to more efficient and competent adoption of SEEK.

**Table 1:** SEEK training strategies

SEEK MOC-4	Independent online (IND)
Eligible for MOC-4 and CME credits	Eligible for MOC and CME credits
Engage in SEEK QI project—PDSA cycle	N/A
Participate in four 1-hr webinars	N/A
View SEEK training videos (2.5 h)	View SEEK training videos
Pass SEEK post-test	Pass SEEK post-test
Participate in 3 1-hr consultation sessions	Participate in 3 1-hr consultation sessions

### Implementation strategies

SEEK*online* is software to efficiently implement SEEK via a secure web-based system interfacing with a practice’s electronic health record (EHR). SEEK*online* has been developed and currently is being beta tested. Its effectiveness in improving implementation outcomes compared to the Traditional paper-and-pencil approach has yet to be examined. SEEK*online* enables parents to privately complete the SEEK PQ-R before a child’s checkup. Responses are available to PCPs at the start of the visit, and there is real-time decision support for PCPs. Selected documentation is sent to the child’s EHR. We anticipate that some practices will choose the Traditional approach over the software (see Table 2).

**Table 2:** SEEK intervention strategies

SEEK*online*	Traditional
Parent completes SEEK PQ online	Parent completes SEEK PQ with paper and pencil
SEEK PQ-R adds probes for positive screens	Probes are conducted during the visit, orally
PCP has parent’s info at start of visit	PCP has parent’s info at start of visit
Electronic decision support for PCP	PCP has SEEK algorithms as Word documents
Auto documentation	PCP needs to document
Info sent to private care portal	N/A
Parent Handouts readily printed	Parent Handouts need to be printed in advance
Information readily integrated into EHR	Information less readily integrated into EHR
Aggregate data readily available for QI projects	Aggregate data not readily available for QI projects

## Methods

### Aims

The overall aims of the proposed study are to examine technology-driven approaches to implementing SEEK and to understand facilitators and barriers regarding its implementation and short-term sustainment, while also examining the effectiveness of these strategies. Doing so will advance knowledge in implementation science related to primary care and the prevention of CM. The randomized type III hybrid design [66] leverages a commitment by 5 major healthcare systems to implement SEEK, enabling a rigorous evaluation of implementation strategies to optimize the adoption and delivery of SEEK in primary care settings, and subsequent prevention of CM: the independent online (IND) [59–61] versus in-depth structured MOC training [62–64]. We will also observe the impact of using the SEEK*online* and the Traditional approach to guide fidelity of model delivery. Further, SEEK will be examined in pediatric and family medicine settings, increasing the generalizability of findings.

#### Aim 1: Evaluate the effectiveness of targeted implementation strategies on the implementation of SEEK in primary care settings

Practices will be randomly assigned to one of two training conditions (IND or MOC). (H1) MOC training will lead to more positive PCP attitudes, comfort level, and competence in addressing risk factors for CM. (H2) Users of SEEK*online* will deliver the intervention more often, achieving a higher rate of penetration, and will report higher levels of provider and parent satisfaction than the Traditional mode of delivery. (H3) MOC training and SEEK*online* will together optimize adoption and sustainment of SEEK.

#### Aim 2: Evaluate the impact of inner context variables (e.g., variation between pediatric and family medicine) on the SEEK implementation process and understand associated barriers and facilitators to successful service start-up and sustainment of SEEK delivery

Using a mixed methods approach, standardized measures of the implementation process (Stages of Implementation Completion (SIC)) and associated cost (Cost of Implementing New Strategies (COINS)) will be integrated with qualitative interview data focusing on barriers and facilitators during implementation from exploration to sustainment. Variations in adoption, model fidelity, and sustainment, and the economic ramifications of the SEEK training and implementation strategies will be examined.

#### Aim 3: Examine the effectiveness of the intervention strategies in preventing CM

CM will be measured via prevalence of ICD-10 codes related to CM obtained from EHRs for all children 0–5 attending the practices. (H1) Incidence of CM will be reduced in practices after implementing SEEK. (H2) Practices randomized to MOC training that successfully implement SEEK will have lower incidences of CM than with the IND approach. We will also observe the influence of SEEK*online* and the Traditional approach on CM rates. Additionally, implementation success will be examined in relation to CM prevalence rates.

### Study design (Fig. 2)

The study uses a rigorous hybrid type III design to examine the effectiveness of technology-driven training strategies to facilitate SEEK’s adoption and implementation in pediatric and family medicine settings, and helping prevent CM. The implementation approach is anchored in four stages of the EPIS framework: Exploration, adoption/Preparation, Implementation, and Sustainment [67].

**Fig. 2:**
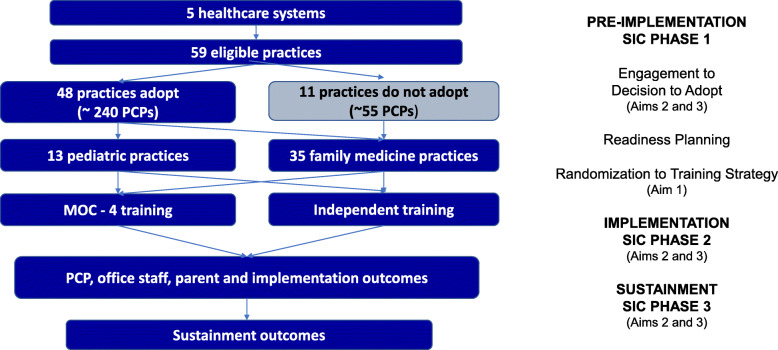
Sequence of steps from pre-implementation (engagement, feasibility, readiness planning) to implementation to sustainment

#### Exploration and sample

Leaders in five healthcare systems that strongly supported the grant application will be approached to formally approve participation, without committing individual practices. They include 59 practices with 306 PCPs. Different regions provide diversity in terms of urban, suburban, and rural locations, and racial/ethnic diversity. Three systems have integrated behavioral health professionals. We plan to study 13 pediatric and 35 family medicine practices. In addition to five system leaders, we will recruit 5 groups of participants: (1) 48 practice leaders, (2) 275 PCPs, (3) 16 behavioral health professionals, (4) 96 ancillary staff, and (5) 288 parents (6 per practice). Smaller subsamples will be purposively selected for qualitative interviews according to practice type, training strategy, and presence of integrated behavioral health.

Inclusionary criteria are as follows: (1) practices not already implementing SEEK, (2) practices providing primary care to children, and (3) agreement to participate. A letter will be sent to physician leaders of practices inviting participation in the study. If interested, we will hold a 1-hr webinar with the practice leader, PCPs, and behavioral health professionals and key office staff. We will provide written material detailing what their involvement will entail. Participation will be voluntary, and while we prefer that all the PCPs in a practice participate, this will not be required. We anticipate that behavioral health professionals and office staff will necessarily be involved in practices opting to participate. Parents will be recruited via flyers in the waiting area. If interested, they will be asked to notify staff who will request permission to convey their contact information to SEEK project staff. We will contact them and explain the project by phone and in writing.

#### Adoption/preparation

We will sign an MOU with participating practices and request a limited informed consent by practice leaders and PCPs who opt out to gather data influencing their decision. We will identify a physician “champion” and an office staff member to lead implementation in each practice. Practices will be randomized to one of the two training strategies (IND vs. MOC). Practices, however, will be able to select the facilitation strategy (SEEK*online* or Traditional) as we are unable to require this of participants. The design accounts for heterogeneity in geography, size of healthcare systems, type of primary care (pediatric and family medicine), and presence of integrated behavioral health. As shown in Fig. 2, professionals, office staff, and parents are nested within practices which are nested within the 5 healthcare systems. To ease introduction of the SEEK model, we will address logistical issues such as for which checkups parents will complete the SEEK PQ-R and documentation in the EHR, and the SEEK Parent Handouts will be customized with information on local resources.

#### Implementation

Informed consent will be obtained online from all participants at the beginning of the baseline survey. Practice leaders, PCPs, behavioral health professionals and office staff will be asked to complete the surveys (see Table 3) prior to the PCP training. Subsamples will be selected for 30-min phone qualitative interviews. At the end of training, PCPs will evaluate it. Interested practices will have SEEK*online* connected to their EHR, ensuring interoperability. Procedures for addressing concerns of possible CM will be aligned with federal, local, and professional guidelines.

**Table 3:** Study measures related to specific aims

Domain/measure	Aims	Respondent*	Baseline** A: mo 8 B: mo 15	Training A: by mo 9 B: by mo 16	F/U 1 A: mo 20 B: mo 27	F/U 2 A: mo 29 B: mo 36	F/U 3 A: mo 41 B: mo 48
Organizational							
System Demographics	1, 2, 3	SL	●			●	
Evidence-Based Practice Attitude Scale	1, 2, 3	All, except parents	●		●	●	●
Implementation Leadership Scale	1, 2, 3	All, except parents	●		●	●	●
Implementation Climate	1, 2, 3	All, except parents	●		●	●	
Implementation Process							
Stages of Implementation	2, 3	AS	●	●	●	●	●
Adoption							
SEEK Adoption Survey	2, 3	SL, PL, PCP	●				
SEEK Training							
SEEK Training Evaluation	1, 2, 3	PCP		●			
Child Maltreatment							
EHR review***		IT			●		●
Implementation Outcomes							
SEEK PCP Questionnaire	1, 2, 3	PCP	●	●	●		●
SEEK PCP Survey	1, 2, 3	PCP			●	●	●
SEEK Office Staff	1, 2, 3	AS			●	●	●
SEEK Parent View	1, 2, 3	P			●	●	●
Rate of screening	1, 2, 3	EHR	●	●	●	●	●
Receipt of services	1, 2, 3	EHR	●	●	●	●	●
Costs							
Cost of Implementing New Strategies (COINS)	2, 3	PL, AS, PCP		●	●	●	●
General							
Qualitative phone interviews	1, 2, 3	All		●	●	●	●

*SL system leaders, *PL* practice leaders, *PCP* primary care providers, *BHP* behavioral health professionals, *AS* ancillary staff, *P* parents, *IT* = information technology

**A = 1st cohort of healthcare systems and practices, B = 2nd cohort of healthcare systems and practices

***EHRs will be reviewed by IT toward end of study

#### Sustainment

Participants will be assessed at 12, 21, and 33 months post-baseline regarding their thinking, feeling, behavior, and experiences (see Table 3) to help inform understanding of SEEK’s implementation and sustainment. Final surveys and phone interviews will assess participants’ thoughts and plans about continued use of SEEK. Following the grant period, practices that wish to continue utilizing the implementation strategies will be guided through the process of establishing real-world, not-grant funded contracting.

#### Final 12 months

De-identified aggregate data will be abstracted from practices’ EHRs on all children (0–5) for the periods prior to and during SEEK implementation: rate of eligible visits where screening occurred, types of problems identified, types of actions taken, receipt of services, and CM-related diagnoses using ICD-10 codes. This will be facilitated by 4 of the 5 systems using Epic as their EHR and having in-house IT. The data will be analyzed, the final report prepared, and presentations and papers will continue to be developed.

### Measures (see Table 3)

Measurement will include survey-based assessments, targeted qualitative interviews, observational implementation assessment, and EHR data on service delivery of SEEK (e.g., rate of screening) and CM.

### Organizational assessment

Participants will be asked to complete the SEEK Adoption Survey and the following four measures: (1) Practice Demographics Form, (2) the Evidence-Based Practice Attitude Scales measuring attitudes toward adopting EBPs [68], (3) the Implementation Leadership Scale assessing leader support for EBPs within an organization [69], and (4) the Implementation Climate Scale measuring how an organization views new interventions [70]. A subsample of participants will undergo a semi-structured, 30-min phone interview.

### Implementation process and outcomes

The implementation process and outcomes will be measured primarily using the Stages of Implementation Completion (SIC), an observational assessment tool [10, 71, 72]. The SIC has 8 stages, each including subactivities, extending from Engagement with the developers to achievement of practitioner Competency. Sustainment is an ongoing process toward achievement of stage 8. Multiple adaptations of the measure have led to the development of the empirically derived *Universal SIC*, with items found to be relevant and reliably utilized regardless of service sector or population. For the current study, completion of activities will be monitored by the practice manager and collected monthly by the research team, with data entered into the SIC data collection website. Three scores are calculated for each SIC stage. First, the time that a practice takes for a stage is calculated (Duration Score). Second, the proportion of activities completed within a stage is calculated (Proportion Score). Third, the SIC Stage Score marks the final stage that a site reaches. SIC scores are calculated within each of the three implementation phases: pre-implementation (stages 1–3), implementation (stages 4–7), and sustainment (stage 8).

The SEEK Adoption Survey will capture key influences on the decision whether to adopt SEEK, including perceived barriers and strengths. The SEEK PCP Training Evaluation Form will evaluate PCPs’ perceptions of the training approaches and solicit input as to how the training might be improved. The SEEK PCP Questionnaire, used in both SEEK RCTs [39, 40], assesses PCPs’ thinking and practice with regard to addressing the targeted CM risk factors. We will use the SEEK PCP Survey to assess PCPs’ experience implementing SEEK including their perceptions of its relevance, ease of delivery, helpfulness, and training. The SEEK Office Staff Survey will assess staff experiences with SEEK, including their understanding of their roles and perceived competence in implementing the model. The rate of screening for the targeted problems will be abstracted from EHR data, as will be the receipt of services by parents with positive screens. The SEEK Parent View of Child’s PCP, adapted for pediatric practice from the Patient-Doctor Interaction Scale, will be used to assess change in parent’s views of PCPs associated with stage of implementing SEEK [73].

### Child maltreatment

*EHR CM-related Diagnoses* ICD-10 codes accessible through EHRs [74]. De-identified aggregate data will be gathered toward study end for all children 0–5 attending the practices during the study—for up to 2 years prior to and during the study.

### Qualitative assessment: phone interviews

To assess potential barriers and facilitators of the SEEK intervention, we will conduct 30-min semi-structured phone interviews with purposively selected (based on role, practice type, training strategy, and presence of behavioral health) subsamples of 5 groups: system and practice leaders, PCPs, behavioral health professionals, office staff, and parents, at 3 time points. The first 3 groups will be interviewed around the start of the intervention (with a focus on adoption) and 11 and 21 months later (with a focus on implementation and maintenance). The last 2 groups will be interviewed at 11, 20, and 32 months following start of the intervention, after they have experienced SEEK. Interviewers will use a semi-structured guide using the EPIS framework to align questions with stage of implementation [75]. Participants will be asked about their experiences with SEEK, assessment of training and implementation support, challenges in delivering the intervention, and recommendations for addressing the challenges. Interviews will be digitally recorded and professionally transcribed for analysis.

### Measuring costs

The cost analysis will provide estimates of the overall practice-level costs associated with implementing SEEK. Cost measurement will be organized using the Cost of Implementing New Strategies (COINS) framework which provides a structure for measuring and categorizing costs [76]. Implementation costs include all resources used to deliver SEEK. COINS maps onto the SIC, by tracking the costs and resources needed to complete each implementation activity. In SEEK II, PCPs in the intervention arm did not require more time per child than did controls. It thus seems reasonable to apply this to the current study, rather than conduct another time study.

### Data analysis plan

#### General considerations

Standard statistical methods for calculating point estimates, confidence intervals, and *p* values require the assumption of independence. However, due to participants being clustered within healthcare systems and within practices, this assumption cannot be made. To account for the lack of independence among multiple measures within the same system or practice, we will use mixed effects (i.e., hierarchical) models. In these models, we will include random effects for system and for practice. In analyses involving multiple measures from the same person, we will also include a random effect for person. Below, we highlight the main analyses for each aim:

*Aim 1: Compare alternative approaches to implementation of SEEK with respect to clinical and implementation outcomes.* To address aim 1a, we will compare practices randomized to MOC to practices randomized to IND with respect to clinical and implementation outcomes. These include PCPs’ perceptions of the training, scales from the SEEK PCPQ (e.g., competence in addressing problems), and the PCP Survey (e.g., ease of delivery). We will also compare the groups’ rates of screening and parents’ receipt of services and satisfaction with PCPs. Statistical inference will be based on mixed effects models fitted using restricted maximum likelihood. To avoid possible biases due to selective attrition, the primary analysis will follow the “intention to treat” principle including all those randomized. Secondary analyses will be based on groups defined by training received. To address aim 1b, we will examine practices who choose SEEK*online* and those choosing the Traditional approach using the same statistical methods as for aim 1a. Outcomes of interest will include staff satisfaction, costs, rates of screening, receipt of services, and parental satisfaction.

*Aim 2: Examine variations in SEEK’s implementation process and impacts and understand associated barriers and facilitators in pediatric and family medicine practices.* We will determine the proportion of practices that agree to adopt SEEK. We will then assess the relationship between practice characteristics (e.g., demographics, EBP attitudes) and willingness to adopt SEEK. The most important independent predictors will be determined using multivariable logistic regression models. Among those that do adopt SEEK, we will assess their degree of completeness, speed, and quality of implementation using the SIC. Completeness will be summarized using the final stage attained (0–8). Speed will be summarized by the duration in each stage, and quality by the proportion of activities performed at each stage, and overall. The distribution of time to achieve each phase will be estimated using the Kaplan-Meier approach. Barriers and facilitators to implementing SEEK will be probed quantitatively and qualitatively. Among practices adopting SEEK, we will examine the association between practice characteristics and measures of completeness, speed, and quality of implementation from the SIC using multivariable mixed effects models.

*Aim 3: Examine the effectiveness of SEEK in reducing CM.* As in our previous studies [37, 38], we will identify CM-related diagnoses, now via EHRs. We will review the EHR for each child (0–5) in each practice for up to 2 years prior to implementing SEEK and during implementation. The presence of CM-related ICD-10 codes will be recorded. The proportion of children with CM diagnoses before and during SEEK will be compared at each practice. Formal inference regarding the best estimate and statistical significance of pre-post differences will be based on a binary regression model with a random effect for site, similar to aim 1.

#### Cost analysis

Total practice cost of implementation completion will be calculated for both training arms, not for implementing the entire SEEK model. Cost-effectiveness ratios will be calculated as the cost per average SIC component completed, and per average Competence scale and Practice Behavior scale scores. Standard time discounting methods will be applied to cost estimates [77]. We will use sensitivity analyses to derive upper and lower estimates of resource use and implementation costs [77]. Standard errors for use in mean comparisons will be estimated, using bootstrapping methods [77].

#### Qualitative/mixed methods analysis

We will keep an audit trail of data collected and memos, team meetings indicating time, place, source of data, and persons collecting or analyzing information. We will analyze interview transcripts using a thematic content analysis methodology [78]. First, transcripts will be reviewed by investigators to develop a broad understanding of content related to the project’s aims and to identify topics for discussion and observation. Second, segments of text ranging from a phrase to several paragraphs will be assigned codes based on a priori (i.e., from the interview guide) or emergent themes (or open coding) [79]. Codes will be assigned to describe connections between categories and between categories and subcategories (i.e., axial coding) [79]. Codes will also be assigned to reflect participants’ social and demographic characteristics. Lists of codes developed by each investigator will be matched and integrated into a single codebook. Third, each text will be independently coded by at least two investigators. Disagreements in assignment of codes will be resolved through discussion between investigators and by refining definitions of codes. With the final coding structure, two investigators will separately review transcripts to determine level of agreement. A level of agreement ranging from 66 to 97% depending on level of coding (general, intermediate, specific) indicates good reliability in qualitative research [80]. Fourth, based on these codes, the computer program QSR NVivo will generate a series of categories arranged in a tree-like structure connecting text segments grouped into separate categories of codes or “nodes” [81]. These nodes and trees will be used to further the process of axial or pattern coding to examine the association between different a priori and emergent categories. Fifth, by constantly comparing these categories with each other, the different categories will be further condensed into broad themes using a format that places SEEK’s effectiveness and implementation within the framework of the system characteristics [82]. Finally, the themes will be compared with the results of the analysis of quantitative data relating to PCP experiences with SEEK to identify points of convergence and divergence (triangulation) and to explain potentially unanticipated findings (expansion).

## Discussion

CM continues to be a major public health and social problem in all countries. Increasing recognition of the importance of addressing SDH offers an opportunity for scaling up effective EBPs to prevent this problem. This hybrid type III study advances implementation science by applying several theories and established frameworks for evaluating implementation of the effective prevention model, SEEK, in primary care practices. The comparison of two increasingly common technology-driven approaches to medical education should yield valuable information for the SEEK model and other innovations in primary care. The convenience of such modalities is clear; this may be critical for scaling up innovations. While there are options to have such training be interactive, they involve little or no direct human contact, observation and this may be less engaging for students. In addition of how the SEEK*online* software influences implementation should guide related efforts in healthcare. Diverse practices in different parts of the USA are being recruited because these are not controlled settings; each practice has different contextual factors that may influence implementation. The application of the EPIS framework offers a useful model for evaluating the introduction of such interventions in primary healthcare practices.

## Project status

Although delays from the time of grant submission to funding created challenges in recruitment from the originally engaged health systems (due to commonly reported barriers such as changing priorities and resources), 17 practices have been successfully recruited across the USA with the help of several regional and national networks and organizations, such as the American Academy of Pediatrics. In addition, it seemed that practices lacking social work or integrated behavioral health were less inclined to participate. We obtained supplemental funding for a SEEK Helpline to offer consultation to PCPs and staff and help locate local resources, and to offer a modest stipend partly covering practices’ research-related costs. The impact of the COVID-19 pandemic on primary healthcare has however impeded ongoing recruitment efforts and delayed the study.

## Supplementary information

**Additional file 1:** SEEK Protocol Paper – Supplemental Material [83, 84].

## Data Availability

Not applicable; just started gathering data.

## Abbreviations

SEEK: Safe Environment for Every Kid
SSNR: Safe, stable, nurturing relationships
IPV: Intimate partner violence
CM: Child maltreatment
EBP: Evidence-based practice
CPS: Child protective services
MI: Motivational interviewing
PCP: Primary care professional
PQ-R: Parent Questionnaire-R
REAP: Reflect–Empathize–Assess–Plan
QI: Quality improvement
MOC: Maintenance of Certification
IND: Independent (training)
EHR: Electronic health record
SIC: Stages of Implementation Completion
COINS: Cost of Implementing New Strategies
EPIS: Exploration, adoption/Preparation, Implementation, and Sustainment
IT: Information technology
ICD: International Classification of Diseases
SDH: Social determinants of health
