# Supplementary material for: Protocol for comparing two training approaches for primary care professionals implementing the Safe Environment for Every Kid (SEEK) model
Source: Implement Sci Commun. 2020 Sep 22;1:78. doi: 10.1186/s43058-020-00059-9 (PMC7506208; doi:10.1186/s43058-020-00059-9)
Supplement: Supplementary file 1 — Additional file 1: SEEK Protocol Paper – Supplemental Material [83, 84]. [file 43058_2020_59_MOESM1_ESM.docx]

**SEEK Protocol Paper – Supplemental Material**

**Data Analysis Plan**

**General Considerations.** Standard statistical methods for calculating point estimates, confidence intervals, and p-values require the assumption of independence. However, due to participants being clustered within healthcare systems and within practices, this assumption cannot be made. To account for the lack of independence among multiple measures within the same system or practice, we will use random effects (i.e., hierarchical) models. In these models, we will include a random effect for system and for practice. In analyses involving multiple measures from the same person, we will include a random effect for each one. These models can be fitted using SAS Proc GLIMMIX, which fits models for both quantitative and binary outcomes. There will be diverse analyses based on the data. Below we highlight the main analyses for each Aim. The qualitative data (see below) will inform the models through processes of convergence (i.e., triangulation) and expansion (i.e., explanation of unexpected findings from analyses of quantitative data) (83).

Sex as a biological variable will be considered across multiple levels of analyses. At the PCP, parent, and child levels of analyses, there is the potential for variation in implementation outcomes related to sex. Analyses will include a test for sex differences; when differences are noted, additional analyses will examine the influence of additional contextual factors on these outcomes.

**Aim 1.** **Compare alternative approaches to implementation of SEEK with respect to clinical and implementation outcomes.** To address aim 1a, we will compare practices randomized to MOC to practices randomized to IND with respect to clinical and implementation outcomes. These include perceptions of the training from the PCPs’ evaluations, scales from the SEEK PCPQ (e.g., Competence in addressing problems) and the PCP Survey (e.g., Ease of Delivery. We will also compare the groups with respect to rates of screening and parents’ receipt of services and satisfaction with the PCP. For quantitative outcomes, statistical inference will be based on linear regression models; for binary outcomes, inference will be based on logistic regression models, accounting for repeated measures within PCP and system. For example, for PCPs reported Competence we will fit this model: E(Competence_ijkl_) = β_0_ + s_i_ + p_j_ + i_k_ + β_1_(POST) + β_2_(POST)(MOC-4) where Competence_ijkl_ stands for the competence score measured at the lth time point (l=1 or 2 for baseline or follow-up respectively) of the kth PCP in the jth practice of the ith system, and s_i_ , p_j_ , i_k_ are random effects for system, practice and PCP respectively; POST=1 if it is a post-training measure and 0 otherwise, MOC = 1 if the PCP was trained using MOC and 0 if IND, and β’s are parameters to be estimated. Note this model assumes that the mean baseline scores are the same in both groups, as recommended for randomized studies (84). This model can be fitted using restricted maximum likelihood. To avoid possible biases due to selective attrition from the training, the primary analysis will follow the “intention to treat” principle including all those randomized. Secondary analyses will be based on groups defined by training received. To address Aim 1b we will compare practices who choose SEEK-online to practices who choose the traditional approach using the same statistical methods as described for Aim 1a. Outcomes of interest will include staff satisfaction, costs, rates of screening, receipt of services, and parental satisfaction.

**Aim 2. Examine variations in SEEK’s implementation process and impacts and understand associated barriers and facilitators in pediatric and family medicine practices.** We will determine the proportion of practices that agree to adopt SEEK. We will then quantitatively assess the relationship between practice characteristics (e.g., demographics, EBP attitudes) and willingness to adopt SEEK. The most important independent predictors will be determined using multivariable logistic regression models. Among those that do adopt SEEK, we will assess their degree of completeness, speed, and quality of implementation using the SIC. Completeness will be summarized using the final stage attained (0-8). Speed will be summarized by the duration in each stage, and quality by the proportion of activities performed at each stage, and overall. The distribution of these variables will be determined, overall and separately, based on the type of practice. The distribution of time to achieve each phase will be estimated using the Kaplan-Meier approach. Barriers and facilitators to implementing SEEK will be probed quantitatively and qualitatively (see below). Among practices adopting SEEK, we will examine the association between practice characteristics and measures of completeness, speed, and quality of implementation from the SIC. We will examine relationships between practice characteristics and implementation by comparing the distribution of SIC scores in groups defined by practice characteristics. Finally, the most important predictors of degree of implementation will be determined using multivariable mixed effects models with practice characteristics as predictors and SIC measures as outcomes. In addition to studying implementation at the practice level, we will analyze the relationship between PCP level measures of implementation (e.g., screening rates) and PCP characteristics, facilitators and barriers. These models will account for clustering of PCPs within practices, as described above.

**Aim 3. Examine the effectiveness of SEEK in reducing CM.** As in our previous studies, (9,10) we will identify CM-related diagnoses, now via EHRs. We will review the EHR for each child (0-5) in each practice for two years prior to implementing SEEK and during implementation. The presence of CM-related ICD-10 codes will be recorded. The proportion of children with CM diagnoses before and during SEEK will be compared at each practice. Formal inference regarding the best estimate and statistical significance of pre-post differences will be based on a binary regression model with a random effect for site, similarly to Aim 1.

**Sample Size Considerations.** Our projected sample sizes (numbers of practices, professionals within each practice, and children in the practices) should provide good precision to estimate parameters and ample power to detect moderate associations. Table 4 below shows the detectable effect sizes for various comparisons, based on performing 2-sided .05-level tests and incorporate a 1.5-fold increase in variance and sample size to account for potential loss of power due to clustering of observations within practices and systems. In general, the detectable effect sizes are moderate, at plausible levels. We observed effect sizes of about 1.0 regarding Competence and Comfort in SEEK II. For Aim 2, assuming 90% of practices provide organizational information, we will estimate the rate of adoption of SEEK accurately, + 15 percentage points.

Table. Power Calculations.

| **Aim** | **Respondents/Source** | **Measure** | **N per group** | **Detectable Effects with 80% power** |
| --- | --- | --- | --- | --- |
| 1a | PCPs | Changes in Comfort and Competence in addressing CM | 137 | Effect size^1^ of 0.42 |
| 1a | Parents | Parent satisfaction | 144 | Effect size^1^ of 0.41 |
| 1b | Staff | Staff Perceptions | 24/72^2^ | Effect size^1^ of 0.82 |
| 2 | Practices | Proportion adopting | 59 | Precision of +/- 15 pct points |
| 3 | EHR | Pre-post prevalence of CM-related diagnoses | 98,000 | 5% vs. 4.5%^3^  10% vs. 9.6% |

^1^ Defined as the difference in means between groups divided by the standard deviation.

^2^ Conservatively assuming an imbalance between SEEKonline vs. Traditional of as much as 25% to 75%.

^3^ In our previous evaluations of SEEK, we observed rates of CM in the medical record ranging from 5% to 1%.
